# Feasibility and sensitivity study of radiomic features in photoacoustic imaging of patient-derived xenografts

**DOI:** 10.1038/s41598-022-19084-w

**Published:** 2022-09-07

**Authors:** Lorena Escudero Sanchez, Emma Brown, Leonardo Rundo, Stephan Ursprung, Evis Sala, Sarah E. Bohndiek, Ignacio Xavier Partarrieu

**Affiliations:** 1 Department of Radiology, University of Cambridge, Cambridge, CB2 0QQ UK; 2 Cancer Research UK Cambridge Centre, University of Cambridge, Cambridge, CB2 0RE UK; 3 Department of Physics, University of Cambridge, Cambridge, CB3 0HE UK; 4 Cancer Research UK Cambridge Institute, University of Cambridge, Cambridge, CB2 0RE UK; 5 Washington University School of Medicine in St Louis, St. Louis, MO 63110 USA; 6 Department of Information and Electrical Engineering and Applied Mathematics (DIEM), University of Salerno, Fisciano, SA 84084 Italy; 7 Independent Researcher, Cambridge, UK

**Keywords:** Cancer, Cancer imaging, Biomarkers

## Abstract

Photoacoustic imaging is an increasingly popular method of exploring the tumour microenvironment, which can provide insight into tumour oxygenation status and potentially treatment response assessment. Currently, the measurements most commonly performed on such images are the mean and median of the pixel values of the tumour volumes of interest. We investigated expanding the set of measurements that can be extracted from these images by adding radiomic features. In particular, we found that *Skewness* was sensitive to differences between basal and luminal patient derived xenograft cancer models with an $\eta ^2$ of 0.86, and that it was robust to variations in confounding factors such as reconstruction type and wavelength. We also built discriminant models with radiomic features that were correlated with the underlying tumour model and were independent from each other. We then ranked features by their importance in the model. *Skewness* was again found to be an important feature, as were *10th Percentile, Root Mean Squared*, and several other texture-based features. In summary, this paper proposes a methodology to select radiomic features extracted from photoacoustic images that are robust to changes in acquisition and reconstruction parameters, and discusses features found to have discriminating power between the underlying tumour models in a pre-clinical dataset.

## Introduction

Photoacoustic, or optoacoustic, imaging is an emerging imaging modality, currently used in clinical trials^1,2^, which can convey relevant information of the tumour microenvironment^3^. Photoacoustic image contrast arises due to optical absorption, which results in ultrasound generation. When using near-infrared wavelength pulses of light (700–950 nm) for illumination, deoxy- and oxygenated haemoglobin are dominant photoacoustic absorbers of the light, providing readouts of blood content and oxygenation in normal tissues and tumours. Photoacoustic imaging has shown promise in a range of clinical applications, with extensive studies performed in breast and skin cancer diagnosis^1,3–5^.

The high-throughput extraction of features from images, known as radiomics^6–8^, aims to enhance clinical decision making by extracting measurements from images that cannot be perceived by the naked eye. Studies have shown that a radiomics approach can reveal valuable information for disease classification and prognosis; however, it has been found that radiomics results are often difficult to reproduce^9–11^. While radiomics is increasingly utilised in the analysis of structural magnetic resonance images and computed tomography, applications to photoacoustic imaging have so far been limited to studies of ex-vivo patient samples^12,13^. Radiomics for in-vivo photoacoustic imaging warrants further investigation as it may be able to provide real-time additional spatial information relating to heterogeneity in tissue perfusion and texture, in addition to the standard metrics of total haemoglobin and blood oxygenation.

In this paper, we first propose a methodology to enable researchers to determine whether radiomic metrics are sensitive to true variations in the underlying biology or whether they are unduly influenced by variations in the sampling, acquisition or reconstruction steps. We expect such methods to be useful to the wider community, as they will help to characterise the behaviour of radiomics metrics and enable researchers to detect which metrics are most promising for their particular challenge. We then apply these methods to a photoacoustic imaging pre-clinical dataset from murine models and show that, amongst the features selected as the most reliable ones, there is potential to discriminate between two breast cancer patient-derived xenograft (PDX) models, related to two different breast cancer subtypes (basal and luminal B), regardless of the image acquisition and other factors investigated.

## Materials and methods

### Animal models

All animal procedures were conducted in accordance with project and personal licenses, issued under the United Kingdom Animals (Scientific Procedures) Act, 1986 and approved locally by the CRUK Cambridge Institute Animal Welfare and Ethical Review Board under compliance forms CFSB1567 and CFSB1979. There was one basal breast cancer patient-derived xenograft (PDX) model (n = 10) and one luminal B PDX model (n = 11) investigated. All animal methods and results are reported in accordance with the ARRIVE guidelines. There was no control group as the study sought to compare radiomic features in untreated basal and luminal B tumours. Cryopreserved breast PDX tumour fragments in freezing media (heat-inactivated foetal bovine serum (10500064, GibcoTM, Fisher Scientific, Göteborg, Sweden), 10% dimethyl sulfoxide (D2650, Merck)) were kindly donated by the Caldas laboratory at the Cancer Research UK Cambridge Institute (University of Cambridge, CB2 0RE Cambridge, UK). To revive the tissue, these fragments were defrosted at 37 °C, washed with Dulbecco’s modified eagle’s medium (41965039, Gibco) and mixed with matrigel (354262, Corning^®^, NY, USA) before surgical implantation. Tumours were implanted subcutaneously into the flank of 6–9 week-old female NOD scid gamma (NSG) mice (Jax Stock 005557) as per standard protocols^14^. All animals used were acclimatised for 7 days before tumour fragment implantation. Animals were kept in hermetic cages with individual air supply through an EPA filter to guarantee sterile conditions, in 12/12 h ON/OFF light cycles, with enriched environment and food and water ad libitum. Tumour growth was monitored with callipers measuring the diameter along the short and long axes and a mean diameter calculated. Mice were euthanised once the tumour mean diameter reached $\approx$ 1 cm, after their photoacoustic imaging session.

### Photoacoustic imaging

Multispectral Optoacoustic Tomography (MSOT) was used to acquire PA images in a manner similar to previously described protocols15. Briefly, MSOT was performed in the inVision 256-TF scanner (iThera Medical GmBH). The system uses a tunable 660–1300 nm laser. Light is delivered through five fibre bundles to create a near-uniform diffuse illumination beam across the imaging plane. An array of transducers with a centre frequency of 5 MHz (> 55% bandwidth), covering an angle of 270° detects ultrasound waves for tomographic reconstruction. The system has a spatial resolution of approximately 190 µm at 3 cm depth. All data acquisition was performed unblinded. Mice were imaged once tumours reached 1 cm in diameter. Mice were anaesthetised using 3–5% isoflurane. Mice were shaved and depilatory cream applied to prevent hairs introducing image artefacts. Respiratory rate was maintained between 70 and 80 bpm using isoflurane ($\approx$ 1–2% concentration) throughout image acquisition. As described previously^15^, single mice were wrapped in a polyethylene membrane, with ultrasound gel to couple the skin to the membrane and placed into the MSOT system and immersed in water. Water was maintained at 36 °C throughout the procedure. Mice were allowed to stabilise for 15 min before image acquisition with mouse breathing 100% oxygen. The animal holder was translated along the oral-caudal axis of the tumour, with images acquired every 1 mm to capture the tumour volume. Images were acquired using 15 wavelengths between 700 and 880 nm with an average of 6 pulses per wavelength. Each slice took 11.5 s to acquire. An overall imaging session lasted approximately 5 min.

Photoacoustic image reconstruction was performed using both a backprojection and a model-based algorithm in ViewMSOT software (version 3.8, iThera Medical GmBH) over the wavelengths acquired. The backprojection algorithm estimates the initial photoacoustic pressure distribution by the principal of delay-and-sum. The model-based algorithm models the relationship between the initial pressure distribution and the measured photoacoustic signals; the initial pressure distribution is then found iteratively by minimising the squared difference between the modelled signal and the measured signal. Images were reconstructed with a pixel size of 75 µm × 75 µm, which is approximately equal to half of the in-plane resolution of the InVision 256-TF.

### Tumour segmentation

MSOT scans were converted to NIfTI format^16^ using MATLAB (The Mathworks Inc., Natick, MA, USA) version R2019b, and Volumes of Interest (VOIs) were drawn manually around the tumours (excluding the skin). The VOIs were delineated unblinded by the member of the team with experience in mice tumour modelling, using the 3D Slicer software17. All cases were segmented using the images corresponding to the backprojection reconstruction and the wavelength of 800 nm, as this is the isobestic point of oxygenated and deoxygenated haemoglobin. The same delineated VOI was then used to extract radiomic features for the rest of wavelengths and for the model-based reconstruction scans.

**Figure 1:**
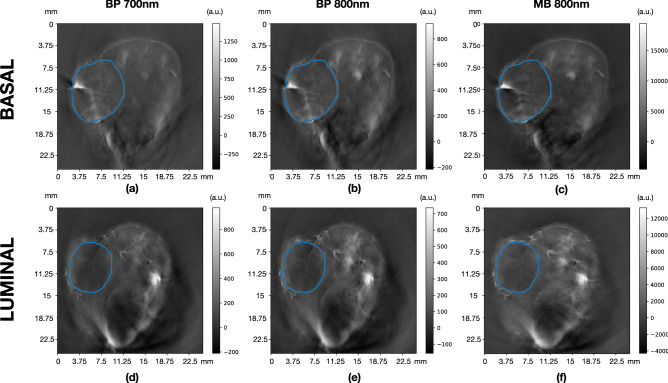
Example 2D photoacoustic images illustrating the volume of interest segmentation performed for one basal PDX (**a**–**c**) and luminal B PDX (**d**–**f**). For each case, images are shown for backprojection (BP) reconstruction, using 700 nm (**a**,**d**) and 800 nm (**b**,**e**) wavelengths, as well as for model-based (MB) reconstruction using 800 nm (**c**,**f**).

Figure 1 shows example 2D photoacoustic images illustrating the segmentation performed for one case of basal (luminal) xenografts on the top (bottom) row. For each case, images are shown for backprojection (BP) reconstruction, using 700 nm (left column) and 800 nm (central column) wavelengths, as well as for model-based (MB) reconstruction using 800 nm (right column). Note that the images in the central column are those used for the manual delineations of the VOIs, then used in the remaining scans.

### Radiomic feature extraction and processing

Radiomic feature extraction and processing was performed blinded, by removing animal model details from the scan names. This was performed by different team members to the one member who drew the VOIs. For the sensitivity analysis a randomly selected luminal B PDX model was removed in order to have a balanced experimental design, which allows for a complete apportioning of the sum of squares. For the five-fold validation we removed 3 luminal B PDX models and 2 basal PDX models randomly, resulting in a balanced design with 8 models of each for the five analyses. For the discriminant analysis all tumours were included. The radiomic features considered in this study were computed using PyRadiomics (version 3.0.1)^17^, a widely used open source Python package for the calculation of such variables.

A total of 93 3D (i.e. calculated from the volume) radiomic features were calculated from the following categories: *first-order statistics* (*FOS*)(18), *Gray Level Co-occurrence Matrix* (*GLCM*)^18–20^ features (24), *Gray Level Dependence Matrix* (*GLDM*)^21^ features (14), *Gray Level Run Length Matrix* (*GLRLM*)^22^ features (16), *Gray Level Size Zone Matrix* (*GLSZM*)^23^ features (16) and *Neighbouring Gray Tone Difference Matrix* (*NGTDM*)^24^ features (5). The full list of features can be found in Supplementary Tables ST1–ST2.

Shape features were discarded from further analysis since all images for each case used the same delineation, regardless of wavelength or reconstruction applied. Thus, no differences between the delineations for a given case exist. Variations in volume between the cases were, however, taken into account, as volume is a well-known intrinsic dependency of some radiomic features^25^, and corrections for such dependencies were implemented as explained in the model discrimination analysis section.

No image filters were applied in the extraction of radiomic features, and the original voxel size of (0.075,0.075,1.0) mm was used. Different quantisations of grey levels (GLs) were used, with the number of GLs being described in Table 1.

### Sensitivity analysis

The initial analysis of the experiments was full factorial, meaning that for each factor all levels were analysed in conjunction with those of the other factors, allowing for a balanced analysis of variance (ANOVA). Tumour model, wavelength, reconstruction type and grey levels were used as factors, with their values described in Table 1. We term factors other than the tumour model *confounding* factors, as they are factors which may obscure differences between models. In order to have a balanced analysis a single randomly selected luminal specimen was left out of the analysis. The sum of squares contributions of these factors and their interactions were compared to the total sum of squares, in order to calculate Pearson’s $\eta ^2$. This serves to describe the variation in the dataset and provides an indication of which factors the radiomic features are most sensitive to. $\eta ^2$ values add up to one, so contributions to feature variance can be clearly apportioned. Radiomics metrics of use would thus be sensitive to the tumour model, but not to other factors investigated. We plotted the $\eta ^2$ contributions as stacked bar charts for ease of visualisation, where we refer to the interactions that may occur between factors as *error*. These were not investigated further due to their relatively small contributions compared to those of individual factors. Groupings of radiomics metrics were separated by a blank space, and a reference table for the features is available in Table ST1 of the supplementary materials.

For factors that were found to dominate the variance, a further analysis was carried out where such factors were standardised. Results were then plotted in a similar manner as before.

Furthermore, a five-fold cross-validation was also performed, where three luminal and two basal specimens were randomly removed from the analyses, resulting in eight of each being analysed. We then re-ran the main effects ANOVA calculations for each of these folds and obtained mean estimates of $\eta ^2$ as well as an associated standard deviation, allowing us to estimate the uncertainty associated with the various $\eta ^2$ contributions, and robustness to sampling effects with the coefficient of variation (CoV). All caculations were done in MATLAB (The Mathworks Inc., Natick, MA, USA) 2018b.

**Table 1:** Factors investigated and their levels

Factor	Levels
Tumour model	Basal (n = 10), Luminal (n = 11)
Wavelength (nm)	700, 730, 750, 760, 770, 800, 820, 840, 850
Grey levels	8, 16, 32, 64, 128, 256
Reconstruction type	Backprojection, Model Linear+

A randomly selected luminal model was left out of the analysis to get a balanced design. The result was 2160 VOIs for analysis.

### Model discrimination analysis

The aim of the model discrimination analysis is to understand if any first or higher order radiomic features could be used to classify the two different models tested: basal and luminal. Before training a machine learning model for classification of the underlying PDX model, a reduction of the number of features was performed. This is a key step, given the large number of radiomic features obtained in comparison to the size of the dataset used. This dimensionality reduction process was performed in two steps: (i) discarding features with no significant correlation with the PDX model and (ii) analysing correlations between the features themselves, selecting amongst those correlated the one with larger individual discriminating power and discarding the others. The specific methods used for each step are described in the following paragraphs. All of the available tumour samples were used for the discrimination analysis.

A preliminary step was implemented to investigate whether the distribution of VOIs was very different between the two tumour models. As some radiomic features present an intrinsic dependency with the tumour VOI, a correction was applied to them following the procedure detailed in previous work^26^.

To analyse correlations between the underlying xenograft model, which is a categorical, non-continuous variable with only two possible values in our study, and each radiomic feature (continuous variables), we computed the Kruskal-Wallis H test^27^ using the Python library *scipy*^28^. The null-hypothesis was that the measurements in all categories came from the same distribution, meaning that when the hypothesis is rejected, a correlation with the model exists. To decrease the false discovery rate, the Benjamini-Hochberg^29^ correction was applied to the Kuskal-Wallis *p*-values, with an allowed false discovery rate of 25%.

Correlations between features themselves were also taken into account. Given the non-independent nature of our measurements, which were taken from the same PDX tumours, varying acquisition parameters and reconstruction algorithms, we used a Repeated Measures Correlation^30^, specifically its implementation in Python through the library *pingouin*^31^.

In addition, we analysed the model classification score achievable with each radiomic feature individually. We fitted three different classifiers, all of them using the scikit-learn Python library^32^: Random Forest Classifier, Gradient Boosting Classifier and Support Vector Machines. A set of default parameter values were chosen. For classifiers based on boosted decision trees, the maximum depth used was 3, with a maximum of 100 estimators and learning rate of 1.0. For SVMs, the selected kernel was Radial Basis Function (rbf), with its hyperparameters set to $\gamma = 0.05$ and $C = 1.0$, and a tolerance of 0.001.

Finally, a random forest classifier was fitted with the selected subset of features resulting from the reduction process. Using the information of the fitted model, the ranking of the features in terms of discriminating power was visualised by constructing SHAP (SHapley Additive exPlanations) plots^33^, using the python implementation^34^. Such plots are based on the Shapley values^35^ from game theory, and allow to sort features by their importance in the model prediction according to their Shapley values, hence providing information about which features are the most important ones when the fitted model makes a prediction on a given case.

## Results

In this study we analysed radiomics features across a pre-clinical photoacoustic imaging dataset of two breast cancer PDX models (one basal and one luminal B). We first analysed the sensitivity of these features to changes in the confounding factors before determining their ability to discriminate between the two PDX models.

### Sensitivity analysis

**Figure 2:**
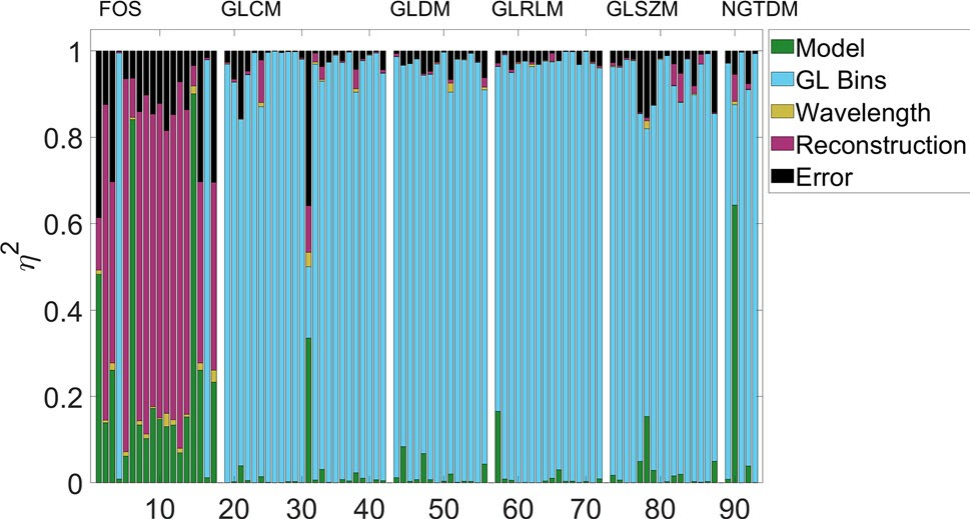
Sensitivity contributions of factors investigated in final radiomics metrics results. First-order statistics are mostly sensitive to variations in the reconstruction type, whereas texture features can be seen to be mostly sensitive to grey level binning variations. Two features, *Skewness* and *Kurtosis*, can be seen to be robust to these changes and mostly sensitive to variations in the tumour model. The feature IDs (x-axis) follows Supplementary Tables ST1 and ST2.

The results of the main effects analysis on the full dataset (minus one luminal specimen) are displayed in Fig. 2. Many radiomics features were more sensitive to changes in the confounding factors investigated than to changes between the two tumour models. First-order statistics, representing the first group of features, were seen to mostly be sensitive to the reconstruction type, apart from two which were sensitive to grey level binning, namely entropy and uniformity. *Skewness* and *Kurtosis*, were robust to variations in the confounding factors and can be assumed to reliably distinguish between tumour models, having an $\eta ^2$ value of above 0.8. Most texture based features on the other hand were strongly sensitive to the binning parameters, which dominate the variance of these features, apart from *NGTDM Coarseness*.

**Figure 3:**
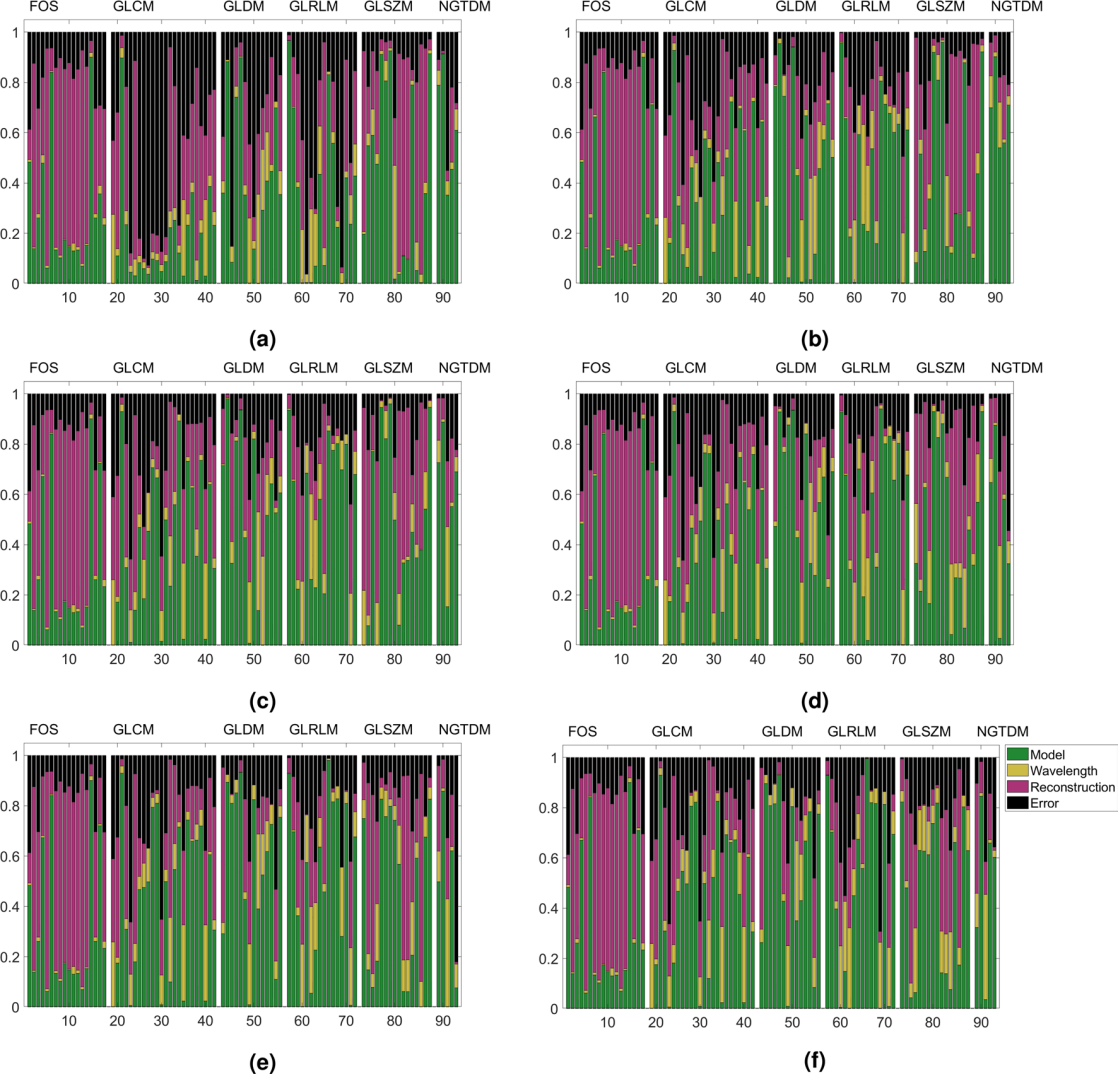
Sensitivity contributions of factors investigated once grey levels have been standardised to (**a**) 8 bins, (**b**) 16 bins, (**c**) 32 bins, (**d**) 64 bins, (**e**) 128 bins and (**f**) 256 bins. The feature IDs (x-axis) follows Supplementary Tables ST1 and ST2.

**Figure 4:**
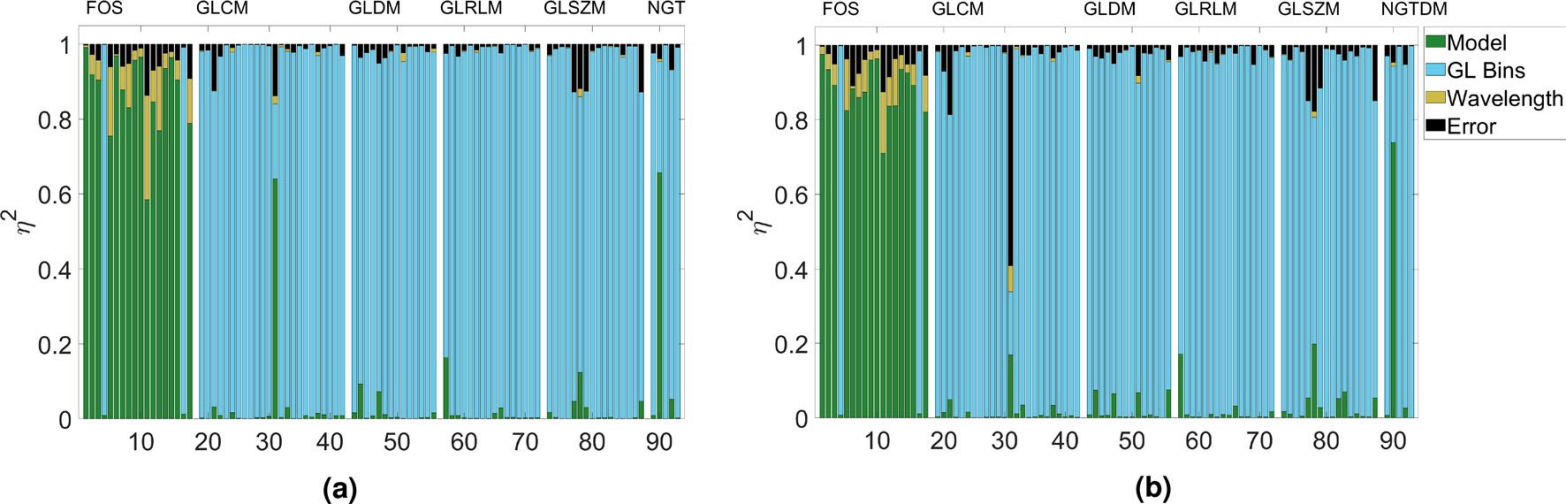
Sensitivity contribution of factors investigated once grey levels have been standardised for reconstruction type where (**a**) is Backprojection and (**b**) is Model linear +.

As features were sensitive to reconstruction type and grey level binning, we decided to implement the analysis standardising for these two factors. The results may be seen in Figs. 3 and 4, where the analysis was standardised for grey levels and reconstruction type, respectively.

When grey levels were standardised (Fig. 3), the variance contribution of the grey level effects disappeared, so the variance contributions are those of the remaining factors varied. This did not affect most first-order statistics as these are not grey level dependent for the most part, but noticeable changes occur for the texture based metrics. Upon grey level standardisation several features were predominantly sensitive ($\eta ^2 > 0.8$) to differences between the tumour models (basal vs. luminal B PDX), though interaction effects can be seen to contribute large amounts to several features, as well as reconstruction effects. There is no clear optimal binning choice for all texture features, but most features perform most poorly with a bin count of 8.

In a similar manner, when the reconstruction method was standardised for, texture metrics were unaffected due to the dominant effect of grey level binning, however first-order statistics become much more model sensitive. In fact most features boast an $\eta ^2 > 0.8$ regardless of model type. Changes in wavelength then become the remaining dominant effect, as could be expected due to the differential absorption of deoxygenated and oxygenated haemoglobin, which provides the image contrast across the wavelengths chosen.

Finally, running the analysis with five folds reveals that the coefficient of variation (CoV) for the $\eta ^2$ value of *Kurtosis* to the model is 0.22, whereas it is 0.08 for the $\eta ^2$ value of *Skewness* to the model, suggesting that although both demonstrate a high sensitivity to the underlying model, *Skewness* may be more robust to variations in the sampling distribution, and thus more generaliseable across datasets. These values may be observed in Table 2. $\eta ^2$ values for the confounding factors are small, and as such their standard deviation and CoV are large in comparison, as would be expected.

**Table 2:** Results of five fold validation for *Kurtosis* and *Skewness*

Kurtosis			Skewness		
Factor	Mean $\eta ^2 \pm \sigma$	CoV	Factor	Mean $\eta ^2 \pm \sigma$	CoV
Model	0.73 ± 0.16	0.22	Model	0.86 ± 0.07	0.08
Grey level Bins	0.00 ± 0.00	N/a	Grey level Bins	0.00 ± 0.00	N/a
Wavelength	0.02 ± 0.02	1.12	Wavelength	0.03 ± 0.02	0.74
Reconstruction	0.15 ± 0.08	0.54	Reconstruction	0.07 ± 0.03	0.42
Error	0.10 ± 0.07	0.70	Error	0.10 ± 0.03	0.57

$\sigma$ is the standard deviation, and due to rounding error CoV values may not match exactly with mean and standard deviation values present. As grey level binning has practicably no effect on these metrics the CoV calculation was not deemed applicable. Mean values are not expected to precisely match with those in Fig. 2 due to the variation in the sampling distribution, but rather to lie within the interval suggested by the standard deviation.

### Model discrimination analysis

**Figure 5:**
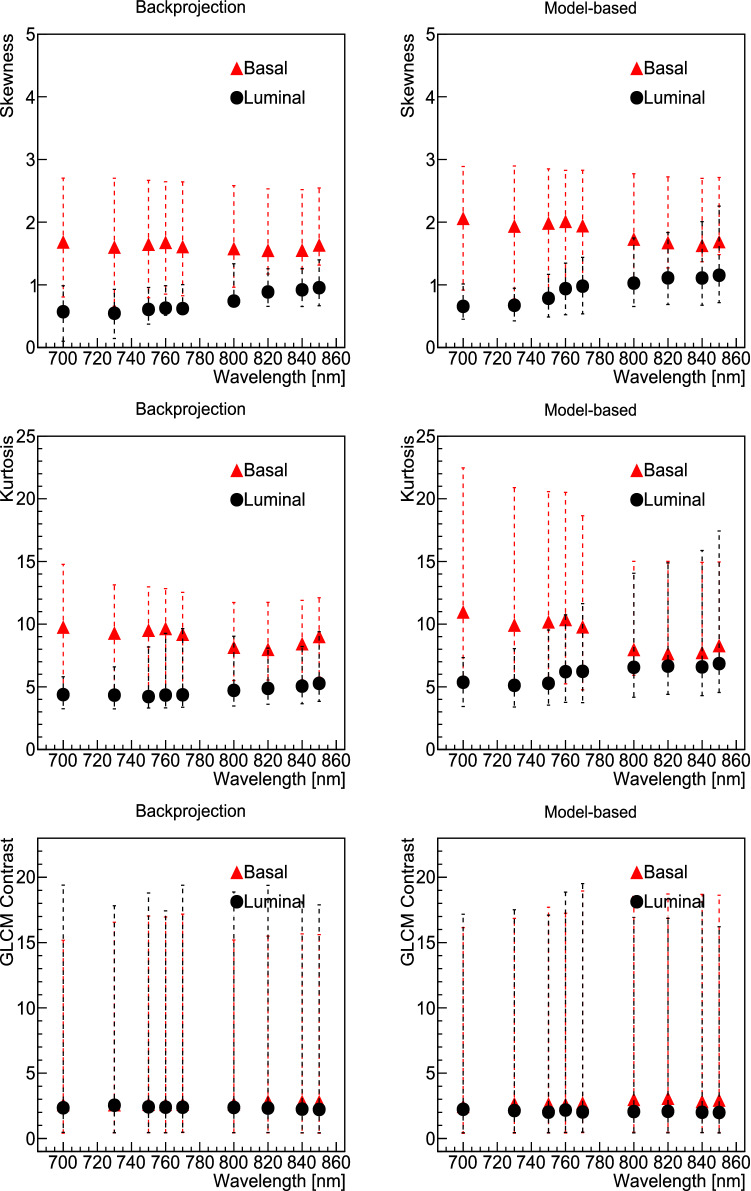
Median (markers) and IQR (lines) of the values of the selected radiomic features: *first-order Skewness* (top row), *first-order Kurtosis* (middle row) and *GLCM Contrast* (bottom row). Values are shown for each model independently, with basal PDX in red and luminal B PDX in blue, as a function of the wavelength used, with backprojection reconstruction on the left and model-based reconstruction on the right. All grey level quantisations were used in each point.

We observed that, regardless of the grey level quantisation used, the *first-order* features of *Kurtosis* and *Skewness* had significant sensitivity to the different tumour models analysed. Figure 5 presents the median and interquartile ranges (IQR) of these two features for each reconstruction and wavelength used. For comparison, a feature in which we observed no sensitivity to the model, the *GLCM* *Contrast*, is also shown in Fig. 5. We observed that in general the *Kurtosis* of the histograms for the basal models were higher than for the luminal model. Similarly, the *Skewness* was higher (and positive) for basal model histograms, whilst it was closer to zero, and in some cases negative, for luminal models. This corresponds with our observations from the histograms with the pixel distributions of each case independently (an example is presented in Supplementary Fig. SF1): basal models appear to have in general a more right-tailed distribution (positive skewness) whilst the distributions for luminal models look more centered, with some examples of left-tailed, and in general a more Gaussian distribution.

Given that the distribution of volumes between the VOIs of tumours corresponding to each model was different (see Supplementary Fig. SF2), we performed a pre-processing step in which we corrected for the volume dependency of some radiomic features. Examples of the values of the features as a function of the size of the VOI, in terms of number of voxels, is presented in Supplementary Fig. SF3 before (left, red) and after (right, blue) corrections are applied.

The Kruskal-Wallis test was performed on the corrected values of each radiomic feature to test the correlation with the model (categorial variable with values basal and luminal). The resulting *p*-value per feature is presented in Supplementary Fig. SF4. In this figure, the horizontal line represents the critical value, corrected following the Benjamini-Hochberg approach, that can be used to differentiate features correlated (below) versus non-correlated (above) with the model.

To further understand the contributions of the radiomic features to model classification, we first reduced the number of features based on their correlations. We eliminated the 24 features that do not appear to have a significant correlation with the model using Kruskal-Wallis *p*-values and the Benjamini-Hochberg correction. Those features are listed in Supplementary Table ST3.

Afterwards, we compared the pair-wise correlations and for each pair that was highly correlated ($>0.9$), we chose the feature with the highest model classification score on its own. The values of the pair-wise correlations of the radiomic features are presented in Supplementary Fig. SF5. Results of the model classification score with each radiomic feature independently are presented in Supplementary Tables ST4 and ST5.

A total of 27 features were selected in this way:

- first-order: 10 Percentile, 90 Percentile/RMS (A tie occurs between these two highly correlated variables, therefore choosing each one of them should be equivalent), Entropy, Kurtosis, Minimum, Skewness.
- GLCM Cluster Prominence, Cluster Shade, Idmn, Idn, *mc2*, MCC, Sum Squares.
- GLDM Dependence Non Uniformity Normalized, Large Dependence Emphasis, Small Dependence High Gray Level Emphasis.
- GLRLM Gray Level Non Uniformity, Gray Level Non Uniformity Normalized, Gray Level Variance, Low Gray Level Run Emphasis.
- GLSZM Gray Level Non Uniformity, Large Area Emphasis, Low Gray Level Zone Emphasis, Size Zone Non Uniformity Normalized.
- NGTDM Busyness, Coarseness, Strength.

**Figure 6:**
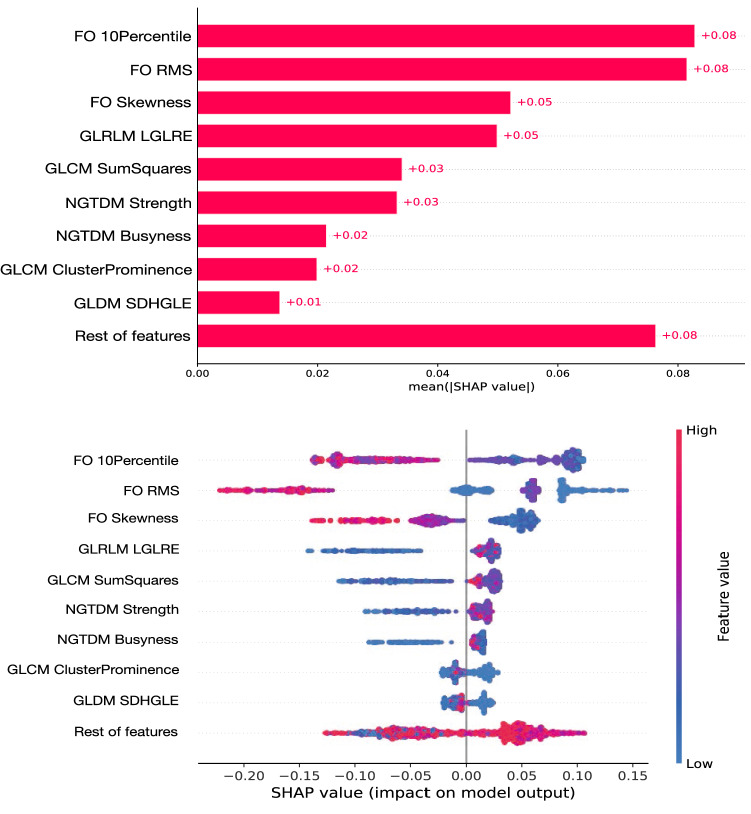
SHAP plots showing the ranking of the top features contributing to a classification model using random forest and the luminal model as signal. The top panel presents a bar chart of the average SHAP value magnitude, as an indicator of the global feature importance, ordered from the one with the highest discriminating power on top. The bottom panel shows the beeswarm distributions of the selected top features in the ranking (top panel), where each dot corresponds to an individual measurement, positioned along the x-axis according to the impact that the feature considered had on the model’s prediction for that specific tumour. Colour in the bottom plot indicates grading between lower and higher values of each radiomic feature.

Finally, with those 27 selected features we fitted a random forest classifier and constructed SHAP plots to show the individual importance and ranking of the features in the model’s prediction. This is presented in Fig. 6, using the model luminal B PDX model as the signal to be selected. This figure is divided in two plots: the top panel presents a bar chart of the average SHAP value magnitude, as an indicator of the global feature importance, order such that the features of highest importance appear at the top; the bottom panel shows the beeswarm distributions of the selected top features, with a dot corresponding to each individual measurement (i.e. dots appear in this plot for the values of each feature that have been measured in our dataset).

The top features showing the highest importance according to the SHAP plots were:

- First-order 10 Percentile
- First-order Root Mean Squared
- First-order Skewness
- GLRLM Low Gray Level Run Emphasis (LGLRE)
- GLCM Sum Squares
- NGTDM Strength
- NGTDM Busyness
- GLCM Cluster Prominence
- GLDM Small Dependence High Gray Level Emphasis (SDHGLE)

As observed, not only histogram-based (first-order statistics) appear within the top 9 features, but also some texture-based features from GLCM, GLDM, GLRLM, and NGTDM.

## Discussion

Radiomics is establishing itself as a method to optimise the extraction of critical diagnostic information in clinical images. However, radiomics metrics have been shown to be quite variable between studies, which has limited their use in the clinic^10^.

We studied the feasibility of using radiomic features in photoacoustic images obtained from patient-derived xenografts, as radiomics has not yet been widely investigated by the photoacoustic imaging community. Previously, photoacoustic images of ex-vivo human prostate samples were processed using texture-based k-means clustering feature learning and demonstrated the potential of these methods to identify prostate biopsy targets^12^.

The main limitation to our analysis was the small sample size of the dataset used. Additional investigations following the methodology suggested here should be carried out with larger datasets to further validate our observations. Power calculations to determine group size could not be performed in the first instance due to absence of previous data using these animals models, imaging modality and radiomic analyses, therefore the group size was based on our previous experience conducting in vivo photoacoustic imaging studies in cell-line models^36^. We used *η*^2^ as a descriptive, not predictive, statistic to demonstrate methodology rather than significance. No statements were made in terms of actual discriminating power of individual radiomics features, as values should be validated using a larger dataset, limiting our study to pair-wise comparisons of feature potential instead. In addition, only two tumour models were used in our studies. It would be beneficial to incorporate more breast subtypes in the future, to test the discriminatory power of the identified radiomic features further. In light of these limitations, the work described in this paper should be considered as a feasibility study, describing a methodology to expand the imaging biomarkers currently used in photoacoustic images with some robust and potentially useful radiomic features.

We did not co-register histology slices with the multi-spectral optoacoustic tomography (MSOT) slices analysed in this study, and therefore we cannot draw correlations between histology features and radiomics features. We have previously optimised a protocol to do this and plan to implement this in future work^37^.

In this paper, we develop and propose a methodology that allows interested researchers to quickly determine whether a radiomic feature may be of use or not in their particular case, using the principles of experiment design and sensitivity analysis. We present results as to the effects of varying grey levels, reconstruction method and wavelength on the differentiation between two different tumour models. This analysis determined in our case that the *first-order* features of *Skewness* and *Kurtosis* were robust to variations of grey levels and reconstruction analysis parameters, as well as wavelength chosen during image acquisition, while remaining sensitive enough to discriminate two breast PDX models of two different breast cancer subtypes. Further analysis then showed that *Skewness* was additionally robust to variations in the sampling distribution through a five fold analysis, whereas *Kurtosis* was not. This suggests that *Skewness* could potentially be reliable in a multi-institution study where these three parameters might be different to the ranges investigated, all else being equal. If acquisitions were to be standardised for reconstruction effects, most *first-order* statistics could then become sensitive indicators of tumour model, according to our observations. Similarly, we found that comparing texture-based features between studies where grey levels vary was unreliable, due to the large variations introduced by the grey level choice, as it has been seen in other radiomics studies^38^. However, if the grey level choice is standardised, some features have the potential to become reliable indicators of the underlying tumour model. We found that there was no clear optimum binning for all texture-based features as they mostly vary individually; 8 bins performs generally worst out of all binning levels tested, as it has been found as well in previous work^26^. It should be noted that variations in radiomic feature values due to binning differences can be determined through appropriate fitting of the data if necessary.

Radiomics is yet to be used widely in the photoacoustic imaging field, owing to the wide use of functional metrics such a total haemoglobin and blood oxygenation measurements^36,39,40^. Here, we provide evidence that radiomics analyses of in-vivo photoacoustic images is feasible and yields additional spatial information, which can distinguish different tumour models and breast cancer subtypes. As sharing data and standardisation of photoacoustic imaging increases globally^41^, robust radiomics features may also serve as a tool to compare data from across laboratories and clinics, regardless of variations in image acquisition or other factors. With our limited dataset, we show that reconstruction differences cause larger feature value changes than differences in wavelengths. For future experiments it would be of interest to investigate additional acquisition and reconstruction parameters that commonly vary such as system manufacturer, voxel size and filtering. It would then be possible to order these by relative contributions to the radiomic feature values, as done in the bar charts here. Efforts are ongoing within the photoacoustic community to standardise image acquisition methods^42^, and we believe this study might be useful to such initiatives.

Using the proposed methodology in this paper, we identified Skewness as a metric with good discriminating power between basal and luminal models, despite variations of other factors. In addition, other *first-order* features were identified as having good discriminating power: *10 Percentile* and *90 Percentile/RMS*. In a setting with standardised factor acquisitions these features could be considered useful, however we would like to highlight again the small dataset size and hence the limitation of potential conclusions to be extracted. Other texture-based features also appeared to have discriminating power in this analysis and can be further considered in other datasets acquired in a similar way, taking into consideration their potential lack of robustness in a non-standardised setting. In particular, some *NGTDM* features, *Strength* and *Busyness*, were found to be correlated with the underlying model, as determined with the SHAP explanation of the random forest model, and could be further explored for model classification when standardised for number of grey levels, as they appear to be robust to changes in wavelength and reconstruction. We propose using these methods to identify promising metrics before carrying out predictive studies.

For the purpose of this analysis, we did not consider shape features; however, volume might indeed be a good metric to differentiate models: we observed that basal tumours were in general larger, with a much more spread distribution of volumes compared to luminal. This is expected as basal tumours grow very quickly, compared to luminal ones.

In summary, we demonstrated the feasibility of radiomic features in photoacoustic imaging, that contain additional spatial information potentially useful to differentiate underlying tumour models. We proposed a methodology to test the robustness and sensitivity of such radiomic features, and illustrated it with a set of histogram-based and texture-based features found robust in our study, for consideration in further analyses of photoacoustic imaging.

## Supplementary Information

Supplementary Information.

## Data Availability

The data and code used for the analyses in this paper are available in GitHub: https://github.com/loressa/Photoacoustic_radiomics_xenografts.
