# Publisher Correction: Feasibility and sensitivity study of radiomic features in photoacoustic imaging of patient-derived xenografts

**DOI:** 10.1038/s41598-022-24697-2

**Published:** 2022-11-30

**Authors:** Lorena Escudero Sanchez, Emma Brown, Leonardo Rundo, Stephan Ursprung, Evis Sala, Sarah E. Bohndiek, Ignacio Xavier Partarrieu

**Affiliations:** 1 Department of Radiology, University of Cambridge, Cambridge, CB2 0QQ UK; 2 Cancer Research UK Cambridge Centre, University of Cambridge, Cambridge, CB2 0RE UK; 3 Department of Physics, University of Cambridge, Cambridge, CB3 0HE UK; 4 Cancer Research UK Cambridge Institute, University of Cambridge, Cambridge, CB2 0RE UK; 5 Washington University School of Medicine in St Louis, St. Louis, MO 63110 USA; 6 Department of Information and Electrical Engineering and Applied Mathematics (DIEM), University of Salerno, Fisciano, SA 84084 Italy; 7 Independent Researcher, Cambridge, UK

Correction to: *Scientific Reports* 10.1038/s41598-022-19084-w, published online 07 September 2022

The original version of this Article contained errors introduced due to miscommunication during the proofing process.

Firstly, the title of the Article, “Feasibility and sensitivity study of radiomic features in photoacoustic imaging of patient-derived xenografts”, was incorrectly given as “Photoacoustic imaging radiomics in patient-derived xenografts: a study on feature sensitivity and model discrimination”.

Secondly, Lorena Escudero Sanchez and Emma Brown were omitted as equally contributing authors.

Thirdly, abbreviations “PAI” and “GL” are now spelled out throughout the Article. As a result, “PAI” now reads as “photoacoustic imaging” and “GL” now reads as “grey level”.

Additionally, in the Materials and Methods section, under the subheading ‘Photoacoustic imaging’,

“An array of transducers covering an angle of 270 °C detects ultrasound waves for tomographic reconstruction.”

now reads:

“An array of transducers with a centre frequency of 5 MHz (>55% bandwidth), covering an angle of 270° detects ultrasound waves for tomographic reconstruction.”

Lastly, some of the content in the Discussion section has been rearranged for readability. Additional qualification has been included to account for the limitations of the study. As a result,

“Radiomics is establishing itself as a method to optimise the extraction of critical diagnostic information in clinical images. However, radiomics metrics have been shown to be quite variable between studies, which has limited their use in the clinic^10^.

We studied the applicability of radiomic features to photoacoustic images obtained from patient-derived xenografts, as radiomics has not yet been widely investigated by the photoacoustic imaging community. Previously, photoacoustic images of ex-vivo human prostate samples were processed using texture-based k-means clustering feature learning and demonstrated the potential of these methods to identify prostate biopsy targets^12^.

In this paper, we develop and propose a methodology that allows interested researchers to quickly determine whether a radiomic feature may be of use or not in their particular case, using the principles of experiment design and sensitivity analysis. We present results as to the effects of varying grey levels, reconstruction method and wavelength on the differentiation between two different tumour models. This analysis determines that the *first-order* features of *Skewness* and *Kurtosis* are robust to variations of grey levels and reconstruction analysis parameters, as well as wavelength chosen during image acquisition, while remaining sensitive to discriminate two breast PDX models of two different breast cancer subtypes. Further analysis then showed that *Skewness* was additionally robust to variations in the sampling distribution through a five fold analysis, whereas *Kurtosis* is not. This suggests that *Skewness* could potentially be reliable in a multi-institution study where these three parameters could be different to the ranges investigated, all else being equal. If acquisitions were to be standardised for reconstruction effects, most *first-order* statistics would then become sensitive indicators of tumour model. Similarly, we found that comparing texture-based features between studies where grey levels vary was unreliable, due to the large variations introduced by the grey level choice, as it has been seen in other radiomics studies^36^. However, if the grey level choice is standardised, several features become reliable indicators of the underlying tumour model. We found that there was no clear optimum binning for all texture-based features as they mostly vary individually; 8 bins performs generally worst out of all binning levels tested, as it was found as well in previous work^26^. It should be noted that variations in radiomic feature values due to binning differences can be determined through appropriate fitting of the data if necessary.

Radiomics is yet to be used widely in the photoacoustic imaging field, owing to the wide use of functional metrics such a total haemoglobin and blood oxygenation measurements^37,38,39^. Here, we provide evidence that radiomics analyses of in-vivo photoacoustic images is feasible and yields additional spatial information, which can distinguish different tumour models and breast cancer subtypes. As sharing data and standardisation of photoacoustic imaging increases globally^40^, robust radiomics features may also serve as a tool to compare data from across laboratories and clinics, regardless of variations in image acquisition or other factors.

Using the proposed methodology in this paper, we identified *Skewness* as a metric with good discriminating power between basal and luminal models, despite variations of other factors. In addition, other *first-order* features were identified as having good discriminating power: *10 Percentile* and *90 Percentile/RMS*. In a setting with standardised factor acquisitions these features could be considered useful. Other texture-based features also appeared to have discriminating power in this analysis and can be further considered in other datasets acquired in a similar way, taking into consideration their potential lack of robustness in a non-standardised setting. In particular, some *NGTDM* features, *Strength* and *Busyness*, were found to be correlated with the underlying model, as determined with the SHAP explanation of the random forest model, and could be further explored for model classification when standardised for number of grey levels, as they appear to be robust to changes in wavelength and reconstruction. We propose using these methods to identify promising metrics before carrying out predictive studies.

For the purpose of this analysis, we did not consider shape features, however, volume might indeed be a good metric to differentiate models: we observed that basal tumours were in general larger, which a much more spread distribution of volumes compared to luminal. This is expected as basal tumours grow very quickly, compared to luminal ones.

The main limitation to our analysis was the small sample size of the dataset used. Additional investigations following the methodology suggested here should be carried out with larger datasets to further validate our observations. Power calculations to determine group size could not be performed in the first instance due to absence of previous data using these animals models, imaging modality and radiomic analyses, therefore the group size was based on our previous experience conducting in vivo PAI studies in cell-line models^38^. We used *η*^*2*^ as a descriptive, not predictive, statistic to demonstrate methodology rather than significance. No statements were made in terms of actual discriminating power of individual radiomics features, as values should be validated using a larger dataset, limiting our study to pair-wise comparisons of feature potential instead. In addition, only two tumour models were used in our studies. It would be beneficial to incorporate more breast subtypes in the future, to test the discriminatory power of the identified radiomic features further.

In summary, we identified a set of histogram-based and texture-based features that could be added to the ones generally used which are robust to image acquisition parameters, reconstruction type and feature extraction choices, and have discriminating power between underlying tumour models captured with photoacoustic imaging.”

now reads:

“Radiomics is establishing itself as a method to optimise the extraction of critical diagnostic information in clinical images. However, radiomics metrics have been shown to be quite variable between studies, which has limited their use in the clinic^10^.

We studied the feasibility of using radiomic features in photoacoustic images obtained from patient-derived xenografts, as radiomics has not yet been widely investigated by the photoacoustic imaging community. Previously, photoacoustic images of ex-vivo human prostate samples were processed using texture-based k-means clustering feature learning and demonstrated the potential of these methods to identify prostate biopsy targets^12^.

The main limitation to our analysis was the small sample size of the dataset used. Additional investigations following the methodology suggested here should be carried out with larger datasets to further validate our observations. Power calculations to determine group size could not be performed in the first instance due to absence of previous data using these animals models, imaging modality and radiomic analyses, therefore the group size was based on our previous experience conducting in vivo photoacoustic imaging studies in cell-line models^36^. We used *η*^2^ as a descriptive, not predictive, statistic to demonstrate methodology rather than significance. No statements were made in terms of actual discriminating power of individual radiomics features, as values should be validated using a larger dataset, limiting our study to pair-wise comparisons of feature potential instead. In addition, only two tumour models were used in our studies. It would be beneficial to incorporate more breast subtypes in the future, to test the discriminatory power of the identified radiomic features further. In light of these limitations, the work described in this paper should be considered as a feasibility study, describing a methodology to expand the imaging biomarkers currently used in photoacoustic images with some robust and potentially useful radiomic features.

We did not co-register histology slices with the nulti-spectral optoacoustic tomography (MSOT) slices analysed in this study, and therefore we cannot draw correlations between histology features and radiomics features. We have previously optimised a protocol to do this and plan to implement this in future work^37^.

In this paper, we develop and propose a methodology that allows interested researchers to quickly determine whether a radiomic feature may be of use or not in their particular case, using the principles of experiment design and sensitivity analysis. We present results as to the effects of varying grey levels, reconstruction method and wavelength on the differentiation between two different tumour models. This analysis determined in our case that the *first-order* features of *Skewness* and *Kurtosis* were robust to variations of grey levels and reconstruction analysis parameters, as well as wavelength chosen during image acquisition, while remaining sensitive enough to discriminate two breast PDX models of two different breast cancer subtypes. Further analysis then showed that *Skewness* was additionally robust to variations in the sampling distribution through a five fold analysis, whereas *Kurtosis* was not. This suggests that *Skewness* could potentially be reliable in a multi-institution study where these three parameters might be different to the ranges investigated, all else being equal. If acquisitions were to be standardised for reconstruction effects, most *first-order* statistics could then become sensitive indicators of tumour model, according to our observations. Similarly, we found that comparing texture-based features between studies where grey levels vary was unreliable, due to the large variations introduced by the grey level choice, as it has been seen in other radiomics studies^38^. However, if the grey level choice is standardised, some features have the potential to become reliable indicators of the underlying tumour model. We found that there was no clear optimum binning for all texture-based features as they mostly vary individually; 8 bins performs generally worst out of all binning levels tested, as it has been found as well in previous work^26^. It should be noted that variations in radiomic feature values due to binning differences can be determined through appropriate fitting of the data if necessary.

Radiomics is yet to be used widely in the photoacoustic imaging field, owing to the wide use of functional metrics such a total haemoglobin and blood oxygenation measurements^36,39, 40^. Here, we provide evidence that radiomics analyses of in-vivo photoacoustic images is feasible and yields additional spatial information, which can distinguish different tumour models and breast cancer subtypes. As sharing data and standardisation of photoacoustic imaging increases globally^41^, robust radiomics features may also serve as a tool to compare data from across laboratories and clinics, regardless of variations in image acquisition or other factors. With our limited dataset, we show that reconstruction differences cause larger feature value changes than differences in wavelengths. For future experiments it would be of interest to investigate additional acquisition and reconstruction parameters that commonly vary such as system manufacturer, voxel size and filtering. It would then be possible to order these by relative contributions to the radiomic feature values, as done in the bar charts here. Efforts are ongoing within the photoacoustic community to standardise image acquisition methods^42^, and we believe this study might be useful to such initiatives.

Using the proposed methodology in this paper, we identified Skewness as a metric with good discriminating power between basal and luminal models, despite variations of other factors. In addition, other *first-order* features were identified as having good discriminating power: *10 Percentile* and *90 Percentile/RMS*. In a setting with standardised factor acquisitions these features could be considered useful, however we would like to highlight again the small dataset size and hence the limitation of potential conclusions to be extracted. Other texture-based features also appeared to have discriminating power in this analysis and can be further considered in other datasets acquired in a similar way, taking into consideration their potential lack of robustness in a non-standardised setting. In particular, some *NGTDM* features, *Strength* and *Busyness*, were found to be correlated with the underlying model, as determined with the SHAP explanation of the random forest model, and could be further explored for model classification when standardised for number of grey levels, as they appear to be robust to changes in wavelength and reconstruction. We propose using these methods to identify promising metrics before carrying out predictive studies.

For the purpose of this analysis, we did not consider shape features; however, volume might indeed be a good metric to differentiate models: we observed that basal tumours were in general larger, with a much more spread distribution of volumes compared to luminal. This is expected as basal tumours grow very quickly, compared to luminal ones.

In summary, we demonstrated the feasibility of radiomic features in photoacoustic imaging, that contain additional spatial information potentially useful to differentiate underlying tumour models. We proposed a methodology to test the robustness and sensitivity of such radiomic features, and illustrated it with a set of histogram-based and texture-based features found robust in our study, for consideration in further analyses of photoacoustic imaging.”

The Supplementary Information published with this Article contained errors. The title of the Article included in the Supplementary Information was updated, and Lorena Escudero Sanchez and Emma Brown were marked as equally contributing authors.

The original Article and accompanying Supplementary Information 1 file have been corrected.

