# Supplementary material for: Feasibility and sensitivity study of radiomic features in photoacoustic imaging of patient-derived xenografts
Source: Sci Rep. 2022 Sep 7;12:15142. doi: 10.1038/s41598-022-19084-w (PMC9452574; doi:10.1038/s41598-022-19084-w)
Supplement: Supplementary file 1 — Supplementary Information. [file 41598_2022_19084_MOESM1_ESM.pdf]

Feasibility and Sensitivity Study of Radiomic Features in Photoacoustic Imaging of Patient-Derived Xenografts

Lorena Escudero Sanchez[1,2,*,†], Emma Brown[3,4,5,†], Leonardo Rundo[1,2,6], Stephan Ursprung[1,2], Evis Sala[1,2], Sarah E. Bohndiek[3,4], and Ignacio Xavier Partarrieu[7,*]

[1]Department of Radiology, University of Cambridge, CB2 0QQ Cambridge, UK
[2]Cancer Research UK Cambridge Centre, University of Cambridge, CB2 0RE Cambridge, UK
[3]Department of Physics, University of Cambridge, CB3 0HE, Cambridge, UK
[4]Cancer Research UK Cambridge Institute, University of Cambridge, CB2 0RE Cambridge, UK
[5]Washington University School of Medicine in St Louis, 63110, MO, USA
[6]Department of Information and Electrical Engineering and Applied Mathematics (DIEM), University of Salerno, 84084 Fisciano (SA), Italy
[7]Independent researcher
[†]These authors contributed equally
[*]Correspondence and requests for materials should be addressed to L.E.S. and I.X.P. (`les44@cam.ac.uk`)

Supplementary Material

List of Figures

SF1 Histograms with the pixel values of the segmented VOIs per case, using as example the backprojection reconstruction and 800nm scans. Blue: basal model; red: luminal model. 6
SF2 Distribution of volumes of the VOIs of tumours corresponding to basal model (left) and luminal model (right). . . 7
SF3 Example of volume correction performed to three features: *firstorder Energy* (top), *GLDM DependenceNonUniformity* (centre) and *NGTDM Coarseness* (bottom). On the left column, values of the features as a function of the number of voxels of the VOI before correction; on the right column, after correction. Red lines represent the function best fitting the data in each case. 8
SF4 p-values of the Kruskal-Wallis test to establish whether each radiomic feature has a correlation with the model (basal vs luminal) or not (null hypothesis). The horizontal line shows the critical value, after the Benjamini-Hochberg correction has been applied to reduce the false positive rate. 9
SF5 Pair-wise correlations (left) and p-values (right) for each pair of radiomic features, computed using repeated measures correlation. The Feature IDs correspond to those in Tables ST1 and ST2. 9

List of Tables

ST1 Feature names and IDs for features corresponding to the categories *first order* and Grey Level Co-occurrence Matrix (*GLCM*). 4

ST2 Feature names and IDs for features corresponding to the categories Grey Level Dependence Matrix (*GLDM*), Grey Level Run Length Matrix (*GLRLM*), Grey Level Size Zone Matrix (*GLSZM*) and Neighbouring Grey Tone Difference Matrix Features (*NGTDM*). 5

ST3 Discarded features after the Kruskal-Wallis test with Benjamini-Hochberg correction. 10

ST4 Scores of three different classifiers, Random Forest, Gradient Boosting and Support Vector Machines, for each radiomic feature individually corresponding to the categories *first order* and *GLCM*. 11

ST5 Scores of three different classifiers, Random Forest, Gradient Boosting and Support Vector Machines, for each radiomic feature individually corresponding to the categories *GLDM*, *GLRLM*, *GLSZM* and *NGTDM*. 12

ID	Feature
1	FO 10Percentile
2	FO 90Percentile
3	FO Energy
4	FO Entropy
5	FO InterquartileRange
6	FO Kurtosis
7	FO Maximum
8	FO MeanAbsoluteDeviation
9	FO Mean
10	FO Median
11	FO Minimum
12	FO Range
13	FO RobustMeanAbsoluteDeviation
14	FO RootMeanSquared
15	FO Skewness
16	FO TotalEnergy
17	FO Uniformity
18	FO Variance
19	GLCM Autocorrelation
20	GLCM ClusterProminence
21	GLCM ClusterShade
22	GLCM ClusterTendency
23	GLCM Contrast
24	GLCM Correlation
25	GLCM DifferenceAverage
26	GLCM DifferenceEntropy
27	GLCM DifferenceVariance
28	GLCM Id
29	GLCM Idm
30	GLCM Idmn
31	GLCM Idn
32	GLCM Imc1
33	GLCM Imc2
34	GLCM InverseVariance
35	GLCM JointAverage
36	GLCM JointEnergy
37	GLCM JointEntropy
38	GLCM MCC
39	GLCM MaximumProbability
40	GLCM SumAverage
41	GLCM SumEntropy
42	GLCM SumSquares

Table ST1. Feature names and IDs for features corresponding to the categories *first order* and Grey Level Co-occurrence Matrix (*GLCM*).

ID	Feature
43	GLDM DependenceEntropy
44	GLDM DependenceNonUniformity
45	GLDM DependenceNonUniformityNormalized
46	GLDM DependenceVariance
47	GLDM GrayLevelNonUniformity
48	GLDM GrayLevelVariance
49	GLDM HighGrayLevelEmphasis
50	GLDM LargeDependenceEmphasis
51	GLDM LargeDependenceHighGrayLevelEmphasis
52	GLDM LargeDependenceLowGrayLevelEmphasis
53	GLDM LowGrayLevelEmphasis
54	GLDM SmallDependenceEmphasis
55	GLDM SmallDependenceHighGrayLevelEmphasis
56	GLDM SmallDependenceLowGrayLevelEmphasis
57	GLRLM GrayLevelNonUniformity
58	GLRLM GrayLevelNonUniformityNormalized
59	GLRLM GrayLevelVariance
60	GLRLM HighGrayLevelRunEmphasis
61	GLRLM LongRunEmphasis
62	GLRLM LongRunHighGrayLevelEmphasis
63	GLRLM LongRunLowGrayLevelEmphasis
64	GLRLM LowGrayLevelRunEmphasis
65	GLRLM RunEntropy
66	GLRLM RunLengthNonUniformity
67	GLRLM RunLengthNonUniformityNormalized
68	GLRLM RunPercentage
69	GLRLM RunVariance
70	GLRLM ShortRunEmphasis
71	GLRLM ShortRunHighGrayLevelEmphasis
72	GLRLM ShortRunLowGrayLevelEmphasis
73	GLSZM GrayLevelNonUniformity
74	GLSZM GrayLevelNonUniformityNormalized
75	GLSZM GrayLevelVariance
76	GLSZM HighGrayLevelZoneEmphasis
77	GLSZM LargeAreaEmphasis
78	GLSZM LargeAreaHighGrayLevelEmphasis
79	GLSZM LargeAreaLowGrayLevelEmphasis
80	GLSZM LowGrayLevelZoneEmphasis
81	GLSZM SizeZoneNonUniformity
82	GLSZM SizeZoneNonUniformityNormalized
83	GLSZM SmallAreaEmphasis
84	GLSZM SmallAreaHighGrayLevelEmphasis
85	GLSZM SmallAreaLowGrayLevelEmphasis
86	GLSZM ZoneEntropy
87	GLSZM ZonePercentage
88	GLSZM ZoneVariance
89	NGTDM Busyness
90	NGTDM Coarseness
91	NGTDM Complexity
92	NGTDM Contrast
93	NGTDM Strength

Table ST2. Feature names and IDs for features corresponding to the categories Grey Level Dependence Matrix (*GLDM*), Grey Level Run Length Matrix (*GLRLM*), Grey Level Size Zone Matrix (*GLSZM*) and Neighbouring Grey Tone Difference Matrix Features (*NGTDM*).

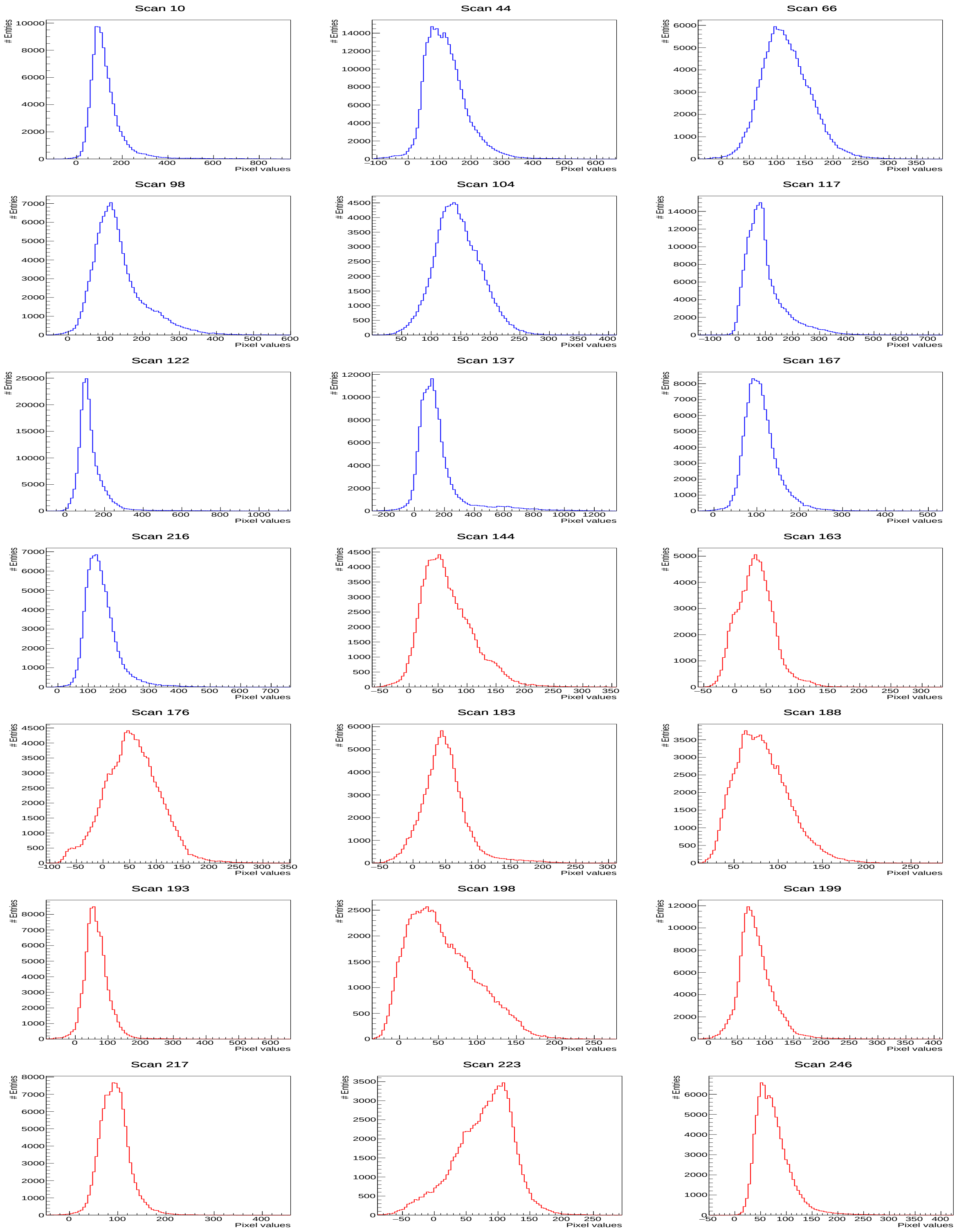

Figure SF1. Histograms with the pixel values of the segmented VOIs per case, using as example the backprojection reconstruction and 800nm scans. Blue: basal model; red: luminal model.

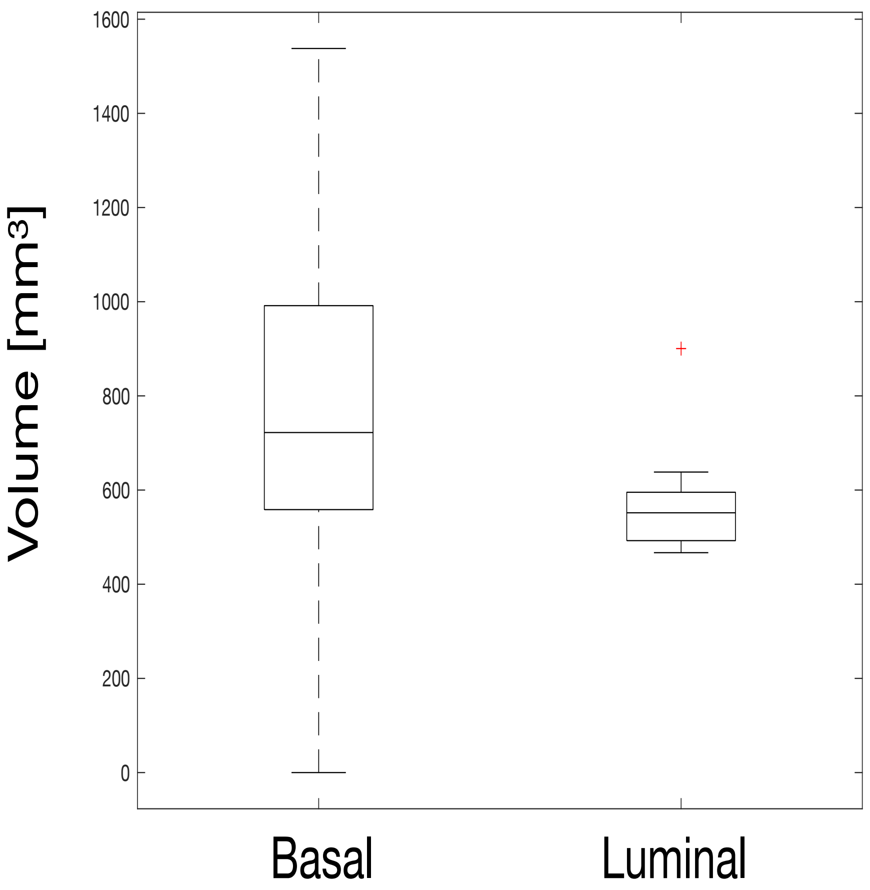

Figure SF2. Distribution of volumes of the VOIs of tumours corresponding to basal model (left) and luminal model (right).

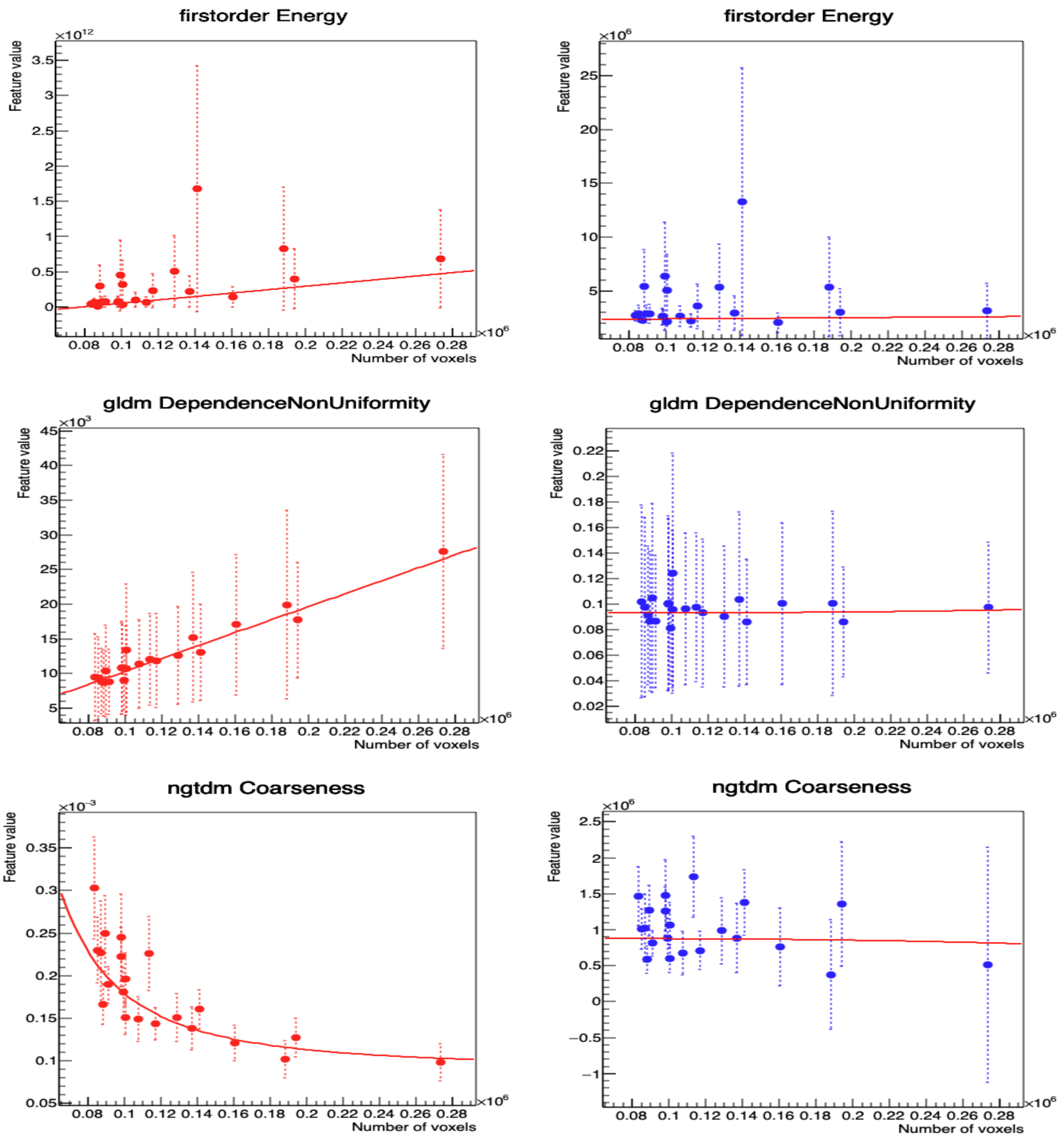

Figure SF3. Example of volume correction performed to three features: *firstorder Energy* (top), *GLDM DependenceNonUniformity* (centre) and *NGTDM Coarseness* (bottom). On the left column, values of the features as a function of the number of voxels of the VOI before correction; on the right column, after correction. Red lines represent the function best fitting the data in each case.

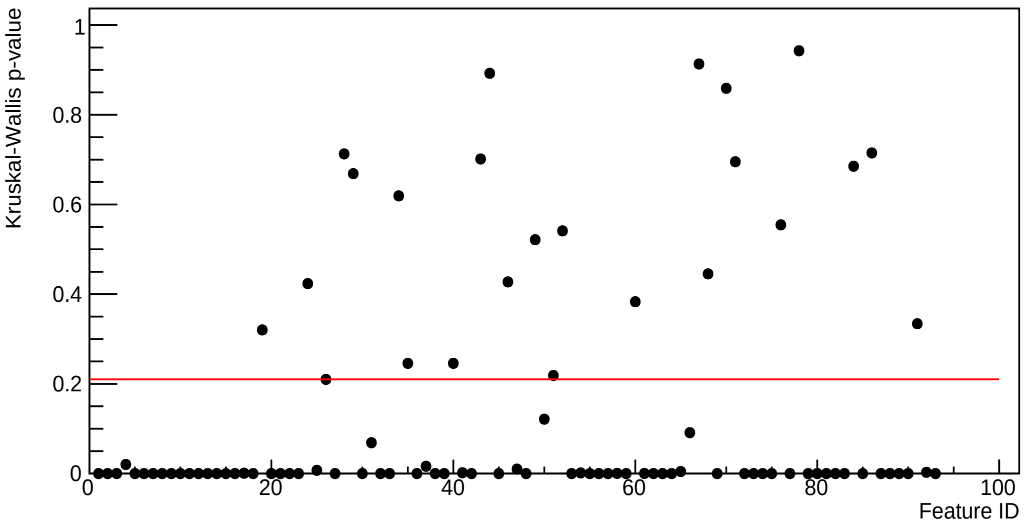

Figure SF4. p-values of the Kruskal-Wallis test to establish whether each radiomic feature has a correlation with the model (basal vs luminal) or not (null hypothesis). The horizontal line shows the critical value, after the Benjamini-Hochberg correction has been applied to reduce the false positive rate.

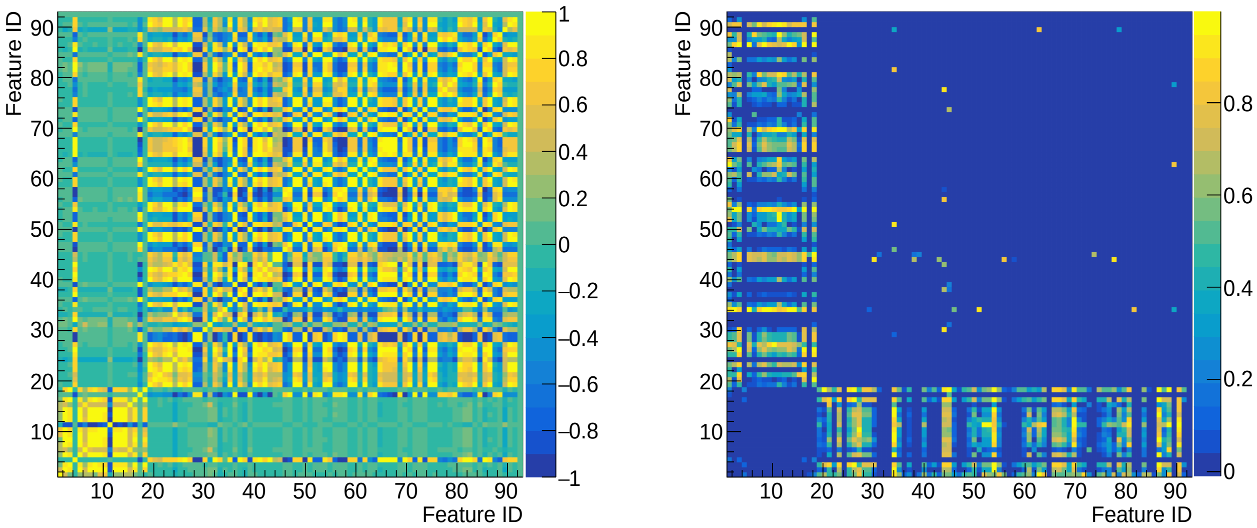

Figure SF5. Pair-wise correlations (left) and p-values (right) for each pair of radiomic features, computed using repeated measures correlation. The Feature IDs correspond to those in Tables ST1 and ST2.

Feature Name	p-value	rank	corrected value
GLCM DifferenceEntropy	0.210	70	0.188
GLDM LargeDependenceHighGrayLevelEmphasis	0.219	71	0.191
GLCM JointAverage	0.246	72	0.194
GLCM SumAverage	0.246	73	0.196
GLCM Autocorrelation	0.320	74	0.199
NGTDM Complexity	0.334	75	0.202
GLRLM HighGrayLevelRunEmphasis	0.383	76	0.204
GLCM Correlation	0.424	77	0.207
GLDM DependenceVariance	0.427	78	0.210
GLRLM RunPercentage	0.445	79	0.212
GLDM HighGrayLevelEmphasis	0.521	80	0.215
GLDM LargeDependenceLowGrayLevelEmphasis	0.541	81	0.218
GLSZM HighGrayLevelZoneEmphasis	0.555	82	0.220
GLCM InverseVariance	0.619	83	0.223
GLCM Idm	0.669	84	0.226
GLSZM SmallAreaHighGrayLevelEmphasis	0.685	85	0.228
GLRLM ShortRunHighGrayLevelEmphasis	0.695	86	0.231
GLDM DependenceEntropy	0.701	87	0.234
GLCM Id	0.713	88	0.237
GLSZM ZoneEntropy	0.715	89	0.239
GLRLM ShortRunEmphasis	0.859	90	0.242
GLDM DependenceNonUniformity	0.892	91	0.245
GLRLM RunLengthNonUniformityNormalized	0.913	92	0.247
GLSZM LargeAreaHighGrayLevelEmphasis	0.943	93	0.25

Table ST3. Discarded features after the Kruskal-Wallis test with Benjamini-Hochberg correction.

ID	Feature	Random Forest	Gradient Boosting	SVM	Average
1	FO 10Percentile	0.823	1.000	0.913	0.912
2	FO 90Percentile	1.000	1.000	1.000	1.000
3	FO Energy	0.894	1.000	1.000	0.965
4	FO Entropy	0.623	0.838	1.000	0.820
5	FO InterquartileRange	0.794	1.000	0.892	0.895
6	FO Kurtosis	0.735	1.000	0.709	0.815
7	FO Maximum	0.802	1.000	0.976	0.926
8	FO MeanAbsoluteDeviation	0.810	1.000	0.873	0.894
9	FO Mean	0.958	1.000	0.987	0.981
10	FO Median	0.944	1.000	0.968	0.971
11	FO Minimum	0.722	1.000	0.894	0.872
12	FO Range	0.799	1.000	0.966	0.922
13	FO RobustMeanAbsoluteDeviation	0.796	1.000	0.862	0.886
14	FO RootMeanSquared	1.000	1.000	1.000	1.000
15	FO Skewness	0.767	1.000	0.675	0.814
16	FO TotalEnergy	0.894	1.000	0.997	0.964
17	FO Uniformity	0.703	0.889	1.000	0.864
18	FO Variance	0.810	1.000	0.995	0.935
19	GLCM Autocorrelation	0.562	0.822	0.731	0.705
20	GLCM ClusterProminence	0.742	0.928	0.524	0.731
21	GLCM ClusterShade	0.567	0.832	0.787	0.728
22	GLCM ClusterTendency	0.764	0.953	1.000	0.906
23	GLCM Contrast	0.703	0.889	0.524	0.705
24	GLCM Correlation	0.601	0.809	0.524	0.645
25	GLCM DifferenceAverage	0.605	0.838	0.524	0.655
26	GLCM DifferenceEntropy	0.562	0.844	0.524	0.643
27	GLCM DifferenceVariance	0.524	0.524	0.524	0.524
28	GLCM Id	0.566	0.819	0.524	0.636
29	GLCM Idm	0.566	0.825	0.524	0.638
30	GLCM Idmn	0.566	0.774	0.524	0.621
31	GLCM Idn	0.590	0.855	0.524	0.656
32	GLCM Imc1	0.524	0.524	0.524	0.524
33	GLCM Imc2	0.524	0.524	0.524	0.524
34	GLCM InverseVariance	0.629	0.840	0.968	0.812
35	GLCM JointAverage	0.581	0.826	0.589	0.665
36	GLCM JointEnergy	0.587	0.810	0.524	0.640
37	GLCM JointEntropy	0.577	0.814	0.535	0.642
38	GLCM MCC	0.609	0.801	0.524	0.645
39	GLCM MaximumProbability	0.644	0.825	0.525	0.665
40	GLCM SumAverage	0.571	0.826	0.608	0.668
41	GLCM SumEntropy	0.557	0.821	0.526	0.635
42	GLCM SumSquares	0.768	0.958	1.000	0.909

Table ST4. Scores of three different classifiers, Random Forest, Gradient Boosting and Support Vector Machines, for each radiomic feature individually corresponding to the categories *first order* and *GLCM*.

ID	Feature	Random Forest	Gradient Boosting	SVM	Average
43	GLDM DependenceEntropy	0.628	0.837	0.996	0.820
44	GLDM DependenceNonUniformity	0.588	0.825	0.524	0.646
45	GLDM DependenceNonUniformityNormalized	0.709	0.896	1.000	0.868
46	GLDM DependenceVariance	0.634	0.853	1.000	0.829
47	GLDM GrayLevelNonUniformity	0.669	0.858	0.877	0.801
48	GLDM GrayLevelVariance	0.524	0.524	0.524	0.524
49	GLDM HighGrayLevelEmphasis	0.564	0.825	0.737	0.709
50	GLDM LargeDependenceEmphasis	0.598	0.836	0.638	0.691
51	GLDM LargeDependenceHighGrayLevelEmphasis	0.717	0.887	1.000	0.868
52	GLDM LargeDependenceLowGrayLevelEmphasis	0.551	0.794	0.556	0.633
53	GLDM LowGrayLevelEmphasis	0.524	0.524	0.524	0.524
54	GLDM SmallDependenceEmphasis	0.636	0.841	0.524	0.667
55	GLDM SmallDependenceHighGrayLevelEmphasis	0.716	0.967	0.524	0.736
56	GLDM SmallDependenceLowGrayLevelEmphasis	0.661	0.844	1.000	0.835
57	GLRLM GrayLevelNonUniformity	0.661	0.850	0.744	0.752
58	GLRLM GrayLevelNonUniformityNormalized	0.737	0.904	1.000	0.881
59	GLRLM GrayLevelVariance	0.717	0.922	1.000	0.880
60	GLRLM HighGrayLevelRunEmphasis	0.567	0.819	0.744	0.710
61	GLRLM LongRunEmphasis	0.594	0.747	0.524	0.622
62	GLRLM LongRunHighGrayLevelEmphasis	0.582	0.794	0.734	0.703
63	GLRLM LongRunLowGrayLevelEmphasis	0.711	0.773	0.524	0.669
64	GLRLM LowGrayLevelRunEmphasis	0.727	0.920	1.000	0.882
65	GLRLM RunEntropy	0.566	0.821	0.524	0.637
66	GLRLM RunLengthNonUniformity	0.649	0.854	0.924	0.809
67	GLRLM RunLengthNonUniformityNormalized	0.575	0.829	0.524	0.643
68	GLRLM RunPercentage	0.593	0.833	0.524	0.650
69	GLRLM RunVariance	0.660	0.762	0.524	0.649
70	GLRLM ShortRunEmphasis	0.571	0.830	0.524	0.642
71	GLRLM ShortRunHighGrayLevelEmphasis	0.589	0.831	0.734	0.718
72	GLRLM ShortRunLowGrayLevelEmphasis	0.731	0.904	1.000	0.878
73	GLSZM GrayLevelNonUniformity	0.745	0.902	1.000	0.882
74	GLSZM GrayLevelNonUniformityNormalized	0.722	0.893	1.000	0.872
75	GLSZM GrayLevelVariance	0.651	0.883	0.524	0.686
76	GLSZM HighGrayLevelZoneEmphasis	0.603	0.840	0.756	0.733
77	GLSZM LargeAreaEmphasis	0.712	0.901	0.524	0.712
78	GLSZM LargeAreaHighGrayLevelEmphasis	0.679	0.872	0.780	0.777
79	GLSZM LargeAreaLowGrayLevelEmphasis	0.748	0.840	0.524	0.704
80	GLSZM LowGrayLevelZoneEmphasis	0.524	0.524	0.524	0.524
81	GLSZM SizeZoneNonUniformity	0.778	0.945	1.000	0.908
82	GLSZM SizeZoneNonUniformityNormalized	0.585	0.813	0.524	0.641
83	GLSZM SmallAreaEmphasis	0.580	0.805	0.524	0.636
84	GLSZM SmallAreaHighGrayLevelEmphasis	0.566	0.840	0.739	0.715
85	GLSZM SmallAreaLowGrayLevelEmphasis	0.729	0.916	1.000	0.882
86	GLSZM ZoneEntropy	0.524	0.524	0.524	0.524
87	GLSZM ZonePercentage	0.665	0.873	0.524	0.687
88	GLSZM ZoneVariance	0.709	0.896	0.524	0.710
89	NGTDM Busyness	0.713	0.925	0.694	0.777
90	NGTDM Coarseness	0.657	0.841	0.999	0.832
91	NGTDM Complexity	0.591	0.849	0.796	0.746
92	NGTDM Contrast	0.573	0.806	0.524	0.634
93	NGTDM Strength	0.760	0.975	1.000	0.912

Table ST5. Scores of three different classifiers, Random Forest, Gradient Boosting and Support Vector Machines, for each radiomic feature individually corresponding to the categories *GLDM*, *GLRLM*, *GLSZM* and *NGTDM*.
